# Glycolysis-cholesterol metabolic axis in immuno-oncology microenvironment: emerging role in immune cells and immunosuppressive signaling

**DOI:** 10.1186/s13578-023-01138-9

**Published:** 2023-10-13

**Authors:** Jing Jin, Qijie Zhao, Zhigong Wei, Keliang Chen, Yonglin Su, Xiaolin Hu, Xingchen Peng

**Affiliations:** 1 Department of Biotherapy, Cancer Center, West China Hospital, Sichuan University, Chengdu, People’s Republic of China; 2 West China School of Medicine, Sichuan University, Chengdu, China; 3 Department of Pharmacy, West China Hospital, Sichuan University, Chengdu, Sichuan People’s Republic of China; 4 Department of Nursing, West China Hospital, Sichuan University, Chengdu, People’s Republic of China; 5 Department of Rehabilitation, Cancer Center, West China Hospital, Sichuan University, Sichuan, People’s Republic of China

**Keywords:** Glycolysis-Cholesterol Metabolic Axis, Immune Cells, Immunosuppressive, Tumor microenvironment (TME)

## Abstract

Cell proliferation and function require nutrients, energy, and biosynthesis activity to duplicate repertoires for each daughter. It is therefore not surprising that tumor microenvironment (TME) metabolic reprogramming primarily orchestrates the interaction between tumor and immune cells. Tumor metabolic reprogramming affords bioenergetic, signaling intermediates, and biosynthesis requirements for both malignant and immune cells. Different immune cell subsets are recruited into the TME, and these manifestations have distinct effects on tumor progression and therapeutic outcomes, especially the mutual contribution of glycolysis and cholesterol metabolism. In particularly, glycolysis-cholesterol metabolic axis interconnection plays a critical role in the TME modulation, and their changes in tumor metabolism appear to be a double-edged sword in regulating various immune cell responses and immunotherapy efficacy. Hence, we discussed the signature manifestation of the glycolysis-cholesterol metabolic axis and its pivotal role in tumor immune regulation. We also highlight how hypothetical combinations of immunotherapy and glycolysis/cholesterol-related metabolic interventions unleash the potential of anti-tumor immunotherapies, as well as developing more effective personalized treatment strategies.

## Introduction

Major biological nodes of cell behavior are controlled by cellular metabolism. Metabolic reprogramming are highly intersecting while providing cells with energy requirements and essential chemical molecules to maintain cell function, proliferation and homeostasis [1]. Metabolic alterations raised the tumor incidence [2]. Noteworthy, tumors undergo numerous metabolic adaptations, and the main feature is changes in glucose and lipid metabolism in the tumor microenvironment (TME) [3, 4]. The immune system was selectively activated to protect against tumor aggression, whereas altered metabolic reprogramming has the potential to dampen their activity and anti-tumor immune response [5]. Emerging evidences indicate that glycolysis and cholesterol metabolism not merely codetermine their differentiation and function, but also metabolic changes in these cells contribute to the cancerization and TME deterioration [6–8].

The most widely studied is aerobic glycolysis or Warburg effect, which demonstrates that cancer cells can accelerate the glycolysis conversion of glucose to lactate even in the presence of adequate oxygen, ultimately meeting the metabolic requirements of proliferating cells [9]. Meanwhile, cholesterol plays an important role in tumorigenesis and regulation of immune responses by its inherent features and modulating signaling pathways [10, 11]. Climbing evidences showed that crosstalk between glycolysis and cholesterol synthesis is a nonnegligible metabolic cascade significant for innate and training immunity [12]. Elucidating the biological mechanisms underlying the glycolysis-cholesterol metabolic axis can promote understanding of tumors, and support the idea that metabolic flexibility rewires tumor to aggressive malignant phenotypes [13].

In this review, we summarized a framework to comprehend the relationship between the glycolysis-cholesterol metabolic axis and immunomodulation, with an emphasis on the relevant immune cell factors involved in disease processes. We also emphasize how hypothetical metabolic interventions that target glycolysis and cholesterol can be used with immunotherapy to maximize the effectiveness of anticancer immunotherapies. Targeting the glycolysis-cholesterol metabolic axis may provide new ideas for individual immunotherapy strategies.

### Metabolic pathogenesis: a non-negligible concept in cancer

Since metabolic reprogramming is an essential aspect of tumorigenesis and immune disorder, some pioneering studies hold that branching point between glycolysis and cholesterol synthesis intersects the cascade in the TME and is deeply involved in immuno-oncology microenvironment [13, 14]. Although there are many clues sword to the glycolysis-cholesterol metabolic axis modulators in inter-/intratumor regulation, the causative or susceptible reasons for them have yet to be identified, especially in the tumor immunity. As a proof-of-concept example, based on gene expression involved in glycolysis and cholesterol synthesis, targeting tumor metabolic plasticity can be used as a means to reprogram an aggressive tumor type [13, 14]. The well-known PI3K/Akt/mTOR cascade activation has an indispensable role in tumor metabolic activities and intracellular biosynthesis [15]. At least in part, the activation permits cell surface nutrient transporter expression and increases uptake of glucose, amino acids, and other nutrients. The enrichment of glycolysis and cholesterol biosynthesis molecular gave the transcriptomic evidences to tumor development and metastasis [16, 17]. Among which, outputs of glycolysis substrate were proved to be involved in the cholesterol production in TME. Moreover, tumor metabolic reprogramming induced glycolysis-cholesterol metabolic axis is required for tumor immunosuppressive cells proliferation and differentiation, and acts as an obstacle for anti-tumor therapy [18]. The glycolysis-cholesterol metabolic axis uncovers energy/lipid-handling capacity, which can lead to nutrient deprivation and cholesterol content regulation, consequently promoting tumor immunosuppressive environment [19]. Glycolysis-cholesterol metabolic axis rewires TME under the control of key regulators, further promoting the tumor development and cells behavior [20]. The statins interference of cholesterol or mevalonate pathway have been proved to be beneficial in patients, but sensitive biomarkers to predict response or underlying mechanisms are still unknown [16]. Notably, glycolysis-cholesterol metabolic axis interconnection determined the cells energy balance and proliferation [21], as well as inhibiting dendritic cells (DCs) and T cell activation in TME and promoting immune evasion [22].

Due to the need of genetic approaches to investigate metabolism of the single cells in TME, current theories and technologies limited mechanistic analysis of glycolysis-cholesterol metabolic axis in impaired tumor immune microenvironment [19]. Specifically, the deteriorating TME presents a highly competitive metabolic environment and extensive byproducts, where some stimuli are involved in the glycolysis and cholesterol cascade as well as tumor immune response [5]. Tumor immunometabolism is tightly implicated in glucose and cholesterol metabolism-related markers, which are also interconnected with branch nodes [4, 23].

Different metabolic gene expression pathways may be actually related to transcriptome-based cancer subtypes, allowing the creation of subtype-specific therapeutic approaches that specifically target metabolic vulnerabilities. Clinical decision-making for therapy selection, possible response prediction, treatment resistance prediction, and likely outcome prediction may be aided by metabolic profiles of malignancies based on metabolic reprogramming.

### Glycolysis-cholesterol metabolic axis in the immune cells of TME

The complex metabolic changes that are present in tumor, where the glycolysis and cholesterol metabolism presented wider implications in the regulation of immune cells response [5, 24]. A deeper understanding of the mechanisms causing metabolic interventions in immune and tumor cells might pave path to novel therapeutic strategies. The glycolysis and cholesterol synthesis axis revealed an important correlation between immune cells and clinical course [17, 24], while climbing evidences implicated it role in tumor immune metabolic regulation. Aerobic glycolysis is the first recognized phenotype of metabolic rewiring in cancer among distinct types of metabolic reprogramming [25]. As a structural element of plasma membranes, cholesterol can adjust membrane fluidity and act as a signal intermediary. Cholesterol homeostasis plays an important role in regulating the immune response, in terms of cell expansion and signaling transduction [26].

### Direct and indirect effects for T cells

**Fig.1:**
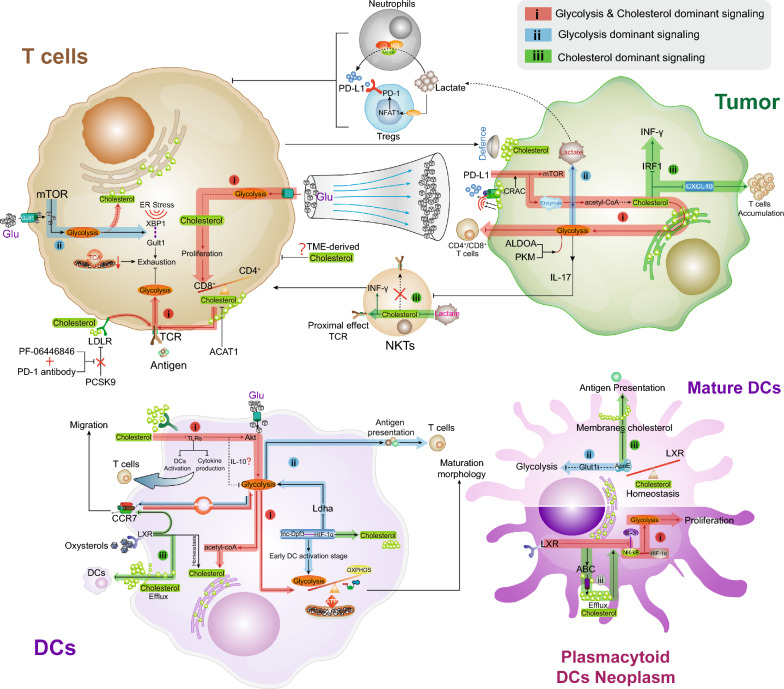
Biological roles of the glycolysis and cholesterol metabolism axis in T cells and DCs-relevant TME. For figure: important glycolysis and cholesterol metabolic crosstalk onto which cells rely are shown in colour; less critical or studied pathways are shown in outside. The glycolysis and cholesterol interconnections exert transinhibition of metabolic signaling in TME-relevant T cells and DCs, and indirectly control neutrophils and Tregs. Scheme of the glycolysis and cholesterol metabolic axis in antitumor immune milieu in response to harsh TME are presented. These factors can perturb proliferation, maturation, activation, migration, or co-stimulatory molecule signaling in T cells and/or DCs. **i** Distant and proximal roles between glycolysis and cholesterol metabolic axis in the TME (Red). Only the undefined or indirect association was marked by dashed lines. **ii** Glycolysis dominant intracellular metabolic signaling to secure energy requirements (Blue). **iii** Cholesterol dominant inner or outer signal transduction and cellular functions (Green). However, several important mechanisms of cholesterol to T cells or IL-10-related DCs metabolism are unclear (denoted by question marks). Each arrow line represented the relationship directly bridge between each other. Glu: Glucose; ER stress: Endoplasmic reticulum stress; TME: Tumor microenvironment; TCR: T-cell receptor; ACAT1: Acetyl-CoA acetyltransferase 1; NKTs: nature killer T cells; LDLR: Low-density lipoprotein receptor; TCA: Tricarboxylic acid cycle; ATP: Adenosine-triphosphate; LXR: Liver X receptors; OXPHOS: Oxidative phosphorylation

The glycolysis-cholesterol metabolic axis regulation will limit T cells functions in the TME [27] (Fig. 1), wherein T cells require metabolites consumption to reach a full effector state [28]. Of note, PD-L1 stimulation is a critical basis of the tumor cell glycolysis, and PD-L1 intervention impairs the tumor glycolysis through the mTOR cascade and glycolysis enzymes [28]. Wang et al. indicated that tumor cell membrane cholesterol contains a cholesterol-recognition amino acid consensus (CRAC) motif, which has the ability to recognize PD-L1, maintain PD-L1 abundance on membrane surface, enhance signaling transduction and prevent molecular degradation [29]. The model of nutrient competition may go beyond glucose metabolism, and glycolysis-cholesterol metabolic axis activation will directly influence T-cell functions in the TME. Increased glycolysis metabolism enhances acetyl-CoA production and critical metabolic intersection, as a feedback, it provided main synthetic raw material for further utilization of cholesterol [30]. The evaluation of membrane signaling-induced tumor glycolysis-cholesterol metabolic axis was proven to dampen T cell-mediated antitumor immunity [31]. Meanwhile, the overexpression of glycolysis genes in tumor cells leaded to irremediable T cells activity exhaustion, although glucose concentrations are restored [32]. High-cholesterol induced tumor glycolysis was potentially accompanied with higher lactate accumulation in the TME [33, 34], where the lactate was proven to restrict T cells response and proliferation [35]. Lactate is an immunomodulatory agent that inhibits immune effector functions, especially T cells, and the lactate signaling potentially serves as a link between metabolism and immunosuppression [36]. As an indirect modulator, lactate could promote the PD-L1 expression on neutrophils via the MCT1/NF-κB/COX-2 cascade, subsequently impairing T-cell effects [37]. In line with this, the immunosuppressive regulatory T cells (Tregs) was reported to absorb lactate through MCT1, and accelerate NFAT1 translocation into the nucleus, thereby promoting the PD-1 expression in Tregs [38]. In compared with effector T cells, Tregs have higher PD-1 expression, and potentially exacerbate glycolysis-cholesterol metabolic axis-mediated TME deterioration [38]. Moreover, higher tumor glycolysis-cholesterol metabolic axis activity was presented with downregulated natural killer T-cells (NKTs) infiltration, wherein interleukin-17 (IL-17) signaling was supposed to be involved in this deleterious process [39, 40]. Intriguingly, NKTs inherent cholesterol was observed to promote T cell receptor (TCR) activation and interferon-γ (IFN-γ) generation, whereas it only influences the proximal TCR signaling rather than distal stimulation [41]. The exogenous lactate will impair NKT function by diminishing the intracellular cholesterol synthesis and IFN-γ production. In addition, in the high glycolysis-cholesterol metabolic axis activated tumor, CXCL10 was potentially to be downregulated and thereby impairing the localization of T cells in TME [31]. A recent study indicated that the cholesterol synthesis was negatively associated with CXCL10 expression during tumor anti-EGFR therapy [42]. Moreover, as a critical transcription factor in the IFN-γ signaling, IFNG-response gene IRF1 was suppressed by accumulated cholesterol [43]. The lower expression of IRF1 in glycolysis-cholesterol metabolic axis activated tumors will impair the tumor IFN-γ signaling functions, thereby dampening the T-cells killing effect [44].

T cells intracellular glycolysis-cholesterol metabolic axis regulation underscores the importance of their direct contribution. Previous study proposed that cholesterol metabolism can regulate the antitumor efficacy of CD8^+^ T cells based immunotherapy [45]. CD8^+^ T cells present with higher plasma membrane cholesterol levels than CD4^+^ T cells, which enhance the TCR multimers, signal transduction, and more efficient immunological synapses formation [46]. In terms of glycolysis-cholesterol metabolic axis feedback regulation, the TCR-based T cells activation signaling could stimulate intracellular glycolysis independent of glucose concentration or the activity of glycolysis enzymes [47]. Activated CD8^+^ T cells had significantly higher cholesterol levels in both the plasma membrane and cytoplasm, and promoted rapid cell proliferation. Genetic ablation or pharmacological inhibition of ACAT1 (a cholesterol esterification enzyme) can restrain cholesterol esterification and shuttling in T cells [48]. As an immune regulatory membrane protein, the TCR complex not merely stimulates T cells glycolysis, but also interacts with cholesterol transporter low-density lipoprotein receptor (LDLR) to further adjust TCR recycling and signaling, ultimately promoting T cells effect [49]. Furthermore, through the control of cholesterol metabolism, HLA gene family were reported to be the important factors that influence the CD8^+^ T cells anti-tumor response [45]. Ma et al. pointed out that extracellular TME-induced cholesterol inhibits CD8^+^ T cells activity and promotes their exhaustion, thus contributing to immune evasion [6]. The accumulation of extracellular ingested cholesterol is specifically connected with CD8^+^ T cells immune checkpoint expression (e.g., PD-1, CTLA-4, TIM-3) and functional exhaustion. Meanwhile, T cells exhaustion was attributed to diminished mitochondrial function and reduced glucose uptake [50], wherein the mTOR signaling and Glut1 expression potentially bridge the glycolysis-cholesterol metabolic axis and lead to glycolysis downregulation [51, 52]. In addition, the endoplasmic reticulum (ER) stress immune checkpoint expression was deemed as another reason for glycolysis-cholesterol metabolic axis mediated CD8^+^ T cells exhaustion. Targeting the ER stress sensor XBP1 or glycolysis/cholesterol-lowering agents showed a great anti-tumor potential in recovering T cells activity [6, 53]. Owing to the plasma membrane of cortically soft cancer cells is rich in cholesterol, T cell-mediated tumor killing will be prevented, whereas hardening tumor cells by depleting cholesterol could enhance T cells cytotoxicity and therapeutic efficacy [54]. Even though, the glycolysis-cholesterol metabolic axis in T cells within TME remains controversial. Precise regulation for the inner and outer regions of effector T cells is important to improve antitumor immunotherapy.

### Dendritic cells relevant regulation in TME

DCs are the basis for initiating and maintaining immune cells responses to tumor. Aggressive tumor types usually present with a severe extracellular milieu, wherein the various signaling aims to dampen the special ability of DCs and impedes the maturation of protective immune responses [55]. The current investigation structured to establish the role of membrane glycolysis-cholesterol metabolic axis in relation to DCs vitality and lifespan [56]. Nevertheless, different subtypes of DCs show heterogeneous manifestations of glycolysis regulation. In compared with conventional DCs (cDC1/2), a lower glucose uptake demand and glycolysis levels are required for merocytic DCs (mcDCs) survival [57].

Toll-like receptors (TLRs) modulate DCs activation and are important for anchoring immune regulation to specific pathogens [58]. Copious extracellular cholesterol concentrations were demonstrated to suppress the DCs cytokine production and activation via TLRs, which consequently deviating the T cells response to hyporesponsive [59]. Importantly, TLR signaling stimulated DCs profoundly enhanced glycolysis, which will orchestrate additional and decisive stimuli for Akt activation, thus boosting the glycolysis rate and cholesterol metabolism [56]. TLR-activated DCs are increasingly reliant on glucose and become more sensitive to death during nutrient limitation. From the point of bioenergetics, TLR signaling induced glycolysis ensures quick supplementation of ATP although less efficiently than oxidative phosphorylation (OXPHOS), eventually underpinning the full maturation morphology and activation of DCs [60]. Herein, the TLR within glycolysis-cholesterol metabolic axis signaling transduction plays a pivotal role in DCs activation. Of note, external stimuli to DCs migration also direct specific immune responses in the TME [61]. Guak et al. reported that glycolysis-cholesterol metabolic axis activation significantly supported the CCR7 aggregation and DCs migration to tumor foci, thereby promoting thrive anti-tumor immune cells, even in the absence of mitochondrial metabolism [62, 63]. In return, CCR7 chemotaxis signal transduction will further evoke a glycolytic response in DCs [64]. Noteworthy, CCR7 is not the sole reason for DCs migration, cell motility is also an important factor. The tumor-derived oxysterols were observed to inhibit DCs surface CCR7 expression by binding with nuclear liver X receptor (LXR), therefore impairing DCs migration and anti-tumor responses [63]. Tumor-derived oxysterols are mainly generated from glycolysis-cholesterol metabolic axis metabolites, such as cholesterol hydroxylase-related oxygenated derivatives [65]. In addition, LXR is involved in intracellular cholesterol homeostasis and promoter regulation, wherein the key and rate-limiting genes in cholesterol metabolism are included [66]. The activated LXR signaling was demonstrated to induce cholesterol efflux from DCs, thereby suppressing DCs activation [67]. On the other hand, exogenous cholesterol accumulation could prevent DCs glycolysis, thereby further decreasing the ingredient acetyl-CoA in cholesterol replenish synthesis, as well as hindering DCs migration and antigen presenting ability [30, 68].

DCs cellular glycolysis-cholesterol metabolic axis signaling transduction underlies the chemotaxis and migration activation [69, 70]. Liu et al. reported that lnc-Dpf3 could directly bind to HIF-1α and suppress the glycolysis gene Ldha transcription, consequently reducing the DCs glycolysis and migration [64]. This process was mainly involved in early glycolysis activation of DCs and provided additional clues for the signal-induced glycolysis-cholesterol metabolic axis response between epigenetic and metabolic mechanisms. Considerable evidence shows that HIF-1α is critical for glycolysis initiation during DCs activation in tumor, which is required for DCs activation and antigen presentation, as well as effector T cells function [71]. Of note, HIF-1α not only influenced glycolysis molecular in DCs, but was also positively associated with stable cellular lipid microenvironment, such as promoting triglyceride and cholesterol accumulation [72]. Intervention with glycolysis marker PKM2, Glut1, and HIF1α contributed to a significant reduction of cholesterol within the DCs [73–75]. Therefore, HIF-1α is an upstream modulator of the glycolysis-cholesterol metabolic axis in DCs. In blastic plasmacytoid DCs neoplasms, the hyperactivation of LXR was supposed to responsible for DCs inhibition and apoptosis, mainly through accelerating adenosine triphosphate–binding cassette (ABC) transporter and apolipoprotein A1-related cholesterol efflux [7]. Meanwhile, hyperactivated LXR will cause the inhibition of NF-kB and IL-3 signaling in DCs, which will be enhanced by cholesterol efflux [7]. NF-kB/HIF-1α cascade downregulation will hinder essential cellular glycolysis and cell proliferation [76]. Herein, crosstalk within glycolysis-cholesterol metabolic axis might further impair the DCs function. In addition, apolipoprotein E (ApoE), an apolipoprotein that involved in glycolysis-cholesterol metabolic axis metabolism, which was tightly linked to DCs membrane cholesterol accumulation. The ApoE deficiency contributed to membrane cholesterol reduction, thereby improving antigen presentation function effects [26]. Meanwhile, the absence of ApoE also showed the ability to promote glycolysis in multipotential progenitor cells and higher Glut1 gene expression [77]. The autocrine/paracrine role of ApoE in controlling cellular cholesterol homeostasis in DCs was superior to LXR [26], suggested that ApoE is another important regulator in glycolysis-cholesterol metabolic axis-mediated DCs immune responses in the TME [78].

### Activation of myeloid-derived suppressor cells

Myeloid-derived suppressor cells (MDSCs) are immature cell heterogeneous populations produced under the pathological situations, which exhibit the immunosuppressive ability to restrain T-cell responses and promote tumor progression [79]. Tumor-derived MDSCs presented with upregulated central carbon metabolism, like glycolysis, pentose phosphate pathway (PPP), and TCA cycles [80], as well as cholesterol synthesis [81]. Due to immune cells dysfunctional or even die when faced with low oxygen tension conditions and scarce glucose supply, high rates of glucose uptake in both tumor cells and MDSCs will facilitate immune evasion and tumor development [79]. Within the TME, MDSCs behavior like tumor cells, which present thriving proliferation and accumulation in most patients [82].

In the nutrient-deprived TME, glycolysis restriction will inhibit granulocyte macrophage colony-stimulating factor (G/GM-CSF) and suppress the MDSCs generation [83]. The high glycolysis rate induced specific CCAAT/enhancer-binding protein beta (CEBPB) and liver-enriched activator protein (LAP) expression via adenosine monophosphate-activated protein kinase (AMPK) autophagy signaling, where the LAP could directly control G-CSF and GM-CSF expression [84]. Upon activation, autophagy-related AMPK signaling simultaneously promotes catabolism and inhibits anabolism [85]. The AMPK-induced cholesterol loss changes tumor lysosomal membrane permeabilization. Herein, energy sensor AMPK was involved in the autophagy-related glycolysis-cholesterol metabolic axis to maintain essential cell viability in nutrient-deprived TME [86]. Moreover, auxiliary evidence indicated that activated CEBPB/PPARG signaling was involved in cholesterol transport [87]. The above tumor cell glycolysis-cholesterol metabolic axis by-products potentially attenuate the anti-tumor immune responses, wherein G-CSF generation could change MDSCs and effector T cells profiles in vivo [84]. Meanwhile, GM-CSF-induced MDSCs presented with upregulated glycolysis gene expression when compared with normal controls, such as glucose transporters Glut1 and PFKl [88]. The GM-CSF-induced MDSCs bring to light that upregulation of intracellular glycolysis limits the reactive oxygen species (ROS) level in a safe range and ensures the MDSCs proliferation and vitality, thereby diminishing the effector T cells response [88]. Of note, with the accumulation of lactic acid dehydrogenase A (LDHA)-induced lactate, G-CSF and GM-CSF will enhance the MDSCs recruitment and immunosuppressive effects to T cells and NKs [8]. Fu et al. indicated that a subtype of MDSCs (CD11b^+^Ly6GlowCD205^+^) was sensitive to glucose metabolism, and interfering the membrane Glut3 significantly shortened its lifespan and was beneficial to antitumor immune response [89]. However, it still unclear how these isoforms are functionally affected by tumors glycolysis-cholesterol metabolic axis signaling molecules in the TME. Nevertheless, in the Epstein-Barr virus (EBV)-induced nasopharyngeal carcinoma, latent membrane protein 1 (LMP1) was demonstrated to increase intrastromal glycolysis and promote MDSCs clonal expansion [80]. In terms of this, with the glycolysis-generated ATP for exosome formation [90], cholesterol-based tumor exosome release promoted the LMP1 endosomal-exosomal transport and signal transduction [91]. In return, LMP1 expression can also increase PI3K and downstream target activation, thereby promoting the glycolysis-cholesterol metabolic axis to enhance LMP1 release [28, 92]. Tumor-derived LMP1 was positively associated with Glut1 expression in membrane of MDSCs, resulting in the promotion of cellular glycolysis to regulate the secretion of the cytokines IL-1β and IL-6 [80]. In parallel, LMP1 interacted with Glut1 will prevent Glut1 protein from K48-ubiquitination and p62-dependent autolysosomal degradation. MDSCs have been demonstrated to have stronger immunosuppressive effect when derived from the tumor milieu [93]. In addition, LXR activation was thought to be responsible for immunosuppressive MDSCs reduction and anti-tumor T cells reactivation in various cancer types [94]. In terms of this, LXR activation upregulates its transcriptional target ApoE, which binds to MDSCs LRP8 synapses to dampen its activity and immunosuppressive function [94]. The hyperactivation of LXR (agonist RGX-104) directly inhibited the MDSCs abundance [95]. In addition to the role of LXR in cholesterol efflux, the endogenous ApoE was thought to be involved in the release of previously synthesized cholesterol than newly synthesized one [96]. Meanwhile, exogenous ApoE was observed to induce the release of both newly and previously synthesized cholesterols [96]. In ApoE^−/−^ mice, MDSCs were significantly decreased [97], suggesting that ApoE enhanced immune suppression via the glycolysis-cholesterol metabolic axis.

Tumor glycolysis-cholesterol metabolic axis-related TME alteration has a significant role in MDSCs accumulation and functions. Different causes of MDSCs dysregulation were associated either as an outer deteriorated milieu and/or inner pathogenic mechanism. LXR-deficient MDSCs contribute to enhanced cell proliferation and expansion [98]. In this aspect, cholesterol-related LXR orchestrates interferon regulatory Factor 8 (IRF-8) transcriptional activation, which is an important negative-regulator for MDSCs differentiation [99]. Moreover, activated IRF-8 is potentially involved in glycolysis-cholesterol metabolic axis processes [45, 100]. LXR/ABCA1-mediated cholesterol efflux plays a nonnegligible role in MDSCs inhibition [101]. Generally, the glycolysis regulator PKM2 in MDSCs was deemed to be negatively associated with LXR and ABCA1 activation, their interaction will promote glycolysis and maintain cholesterol levels to underlie the MDSCs immunosuppressive ability [102, 103]. The upregulation of glycolysis in MDSCs promoted its lifespan and was positively correlated with the content of MDSCs in tumor-bearing individuals. Elevated glycolysis can also prevent MDSCs from producing excessive ROS with an emphasis on glycolysis metabolite phosphoenolpyruvate (PEP), which is an important antioxidant that prevents excessive ROS production, thereby protecting MDSCs from apoptosis [88]. Recently, Hemn et al. observed that the special membrane protein β2-adrenergic receptor (β2-AR) was expressed on MDSCs [104] and may be involved in glycolysis-cholesterol metabolic axis regulation. The stability and activity of β2-AR were markedly associated with cellular membrane cholesterol, sufficient cholesterol could ameliorate β-AR-mediated contractility and Ca2^+^ transients and limit intracellular ROS production [105, 106]. However, the triggering of β2-AR signaling in MDSCs was prefer to decrease glycolysis and promote OXPHOS and fatty acid oxidation (FAO), as well as driving MDSCs immunosuppressive function along with the immunosuppressive mediator PGE_2_ [104].

### Tumor-associated macrophages regulation

**Fig. 2:**
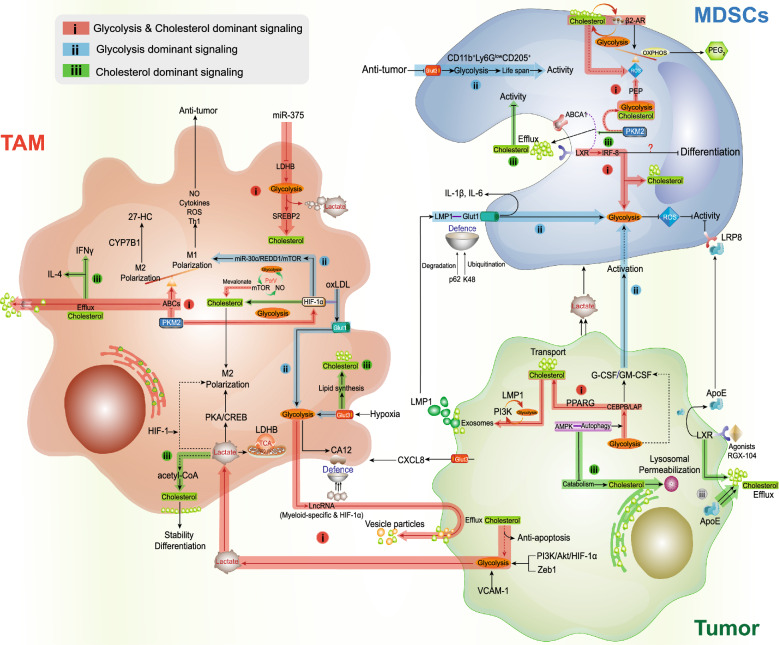
Glycolysis and cholesterol metabolism axis in immunosuppressive TME. Immunosuppressive cells with protumor effects, like TAMs and MDSCs, are recruited and thrive in the TME through tumor metabolic reprogramming relevant effects. The glycolysis and cholesterol metabolism axis and its metabolites have a pivotal role in driving the harsh immunosuppressive tumor milieu, including TAMs and MDSCs. **i** Distant and proximal roles between glycolysis and cholesterol metabolic axis in TME (Red). Only the undefined or indirect association was marked by dashed lines. **ii**: Glycolysis dominant intracellular metabolic signaling to secure cellular energy requirements and activity (Blue). **iii** Cholesterol dominant inner or outer signal transduction and cellular functions (Green). Each arrow line represented the relationship directly bridge between each other. 27-HC: 27-Hydroxycholesterol; LDHB: Lactate dehydrogenase B; NO: Nitric oxide; PGE2: Prostaglandin E2; ROS: Reactive oxygen species

Monocytes are attracted to the tumor location, where they are reprogrammed to become tumor-associated macrophages (TAMs), which are one of the most important components in immunodepression [107]. Attributed to sensitivity to the surrounding TME, TAMs exhibit high levels of functional plasticity and metabolic changes (Fig. 2), such as altered nitrogen cycle metabolism, activation of glycolysis, fatty acid and cholesterol synthesis [108, 109].

The glycolysis-cholesterol metabolic axis intricate metabolic changes between tumor cells and TAMs represent an essential step toward deleterious TME [18]. The increased lactate and other tumor cell byproducts not merely compose the TME, but also drive the TAMs functions [107]. The Glut3 expression, rather than Glut1, is positively connected with glucose uptake, immune cell gathering and immunotherapy response [110]. The lactate from tumor glycolysis could induce macrophages toward an inflammatory pro-tumor phenotype [111]. Among these phenotypes, M2-like TAMs, require internalization of the lactate and rely on HIF-1 stabilization. Moreover, PKA/CREB signaling-induced M2-like TAMs polarization was deemed to be promoted by tumor-derived lactate, which was directly associated with intercellular Zeb1 expression and the PI3K/Akt/HIF-1α activation [112]. Lactate is not sole byproduct of glycolysis, but acts as a fueling and signaling molecule, rescuing exacerbated cells from glucose competition [113]. Notably, as an additional carbon source, lactate is a metabolic and functional regulator of TAMs, which can fuel the TCA cycle in pre-tumor TAMs [114]. The enhanced carbon and acetyl-CoA levels simultaneously increase the cholesterol synthesis, thereby maintaining the cell stability and differentiation [30]. As feedback in glycolysis-cholesterol metabolic axis, highly glycolysis TAMs showed the ability to transmit myeloid-specific long noncoding RNA (lncRNA) and HIF-1α-stabilizing lncRNA to tumor cells [115], which was closely associated with cholesterol efflux mediated vesicle particles [116], consequently enhancing the glycolysis, tumor cells apoptotic resistance and constituting a feed-forward loop.

The differential stimulation of macrophages is supported by profound intracellular metabolic changes, including glycolysis and cholesterol dysregulation [107, 117]. Generally, M1 phenotypic macrophages depend on glycolysis and act as tumor suppressor, while M2 phenotypic macrophages rely on both oxidative metabolism and glycolysis, and been regarded as tumor driver [118]. Evidence has suggested that TAMs have a broad and complex spectrum of polarization, which share characteristics of both M1 and M2 phenotypes, especially the metabolism modulation in their lifespan [119]. TAMs recruitment and tumorigenesis were associated with intracellular glycolysis, and one of the essential instincts of TAMs in the early tumorigenesis is the glycolysis phenotype [120]. Recently, Glut3-overexpressed TAMs were firstly observed to induce glycolysis program in the M2-like polarized phenotype [121]. High Glut3 levels was closely associated with both glucose uptake and lipid synthesis in macrophages, thereby underlying the prerequisite of intracellular glycolysis-cholesterol metabolic axis functions [122]. In parallel, another primary glucose transporter Glut1 in macrophages will induce glycolysis [123], and the absence of Glut1 contributed to TAMs inhibition and tumor regression [124]. Lee et al. reported that cholesterol accumulation modulator oxidized low-density lipoprotein (oxLDL) [125] could promote macrophage recruitment and activation by upregulating Glut1, eventually enhancing macrophages glycolysis [126]. Among which, the alteration of glycolysis-cholesterol metabolic axis metabolism mainly relied on HIF-1α activation, and HIF-1α knockdown completely abrogated the oxLDL functions. On the other hand, crosstalk between oxLDLs and HIF-1α can also promote cholesterol synthesis and M2-like TAMs polarization, as well as decreasing intercellular cholesterol efflux [72]. In inflammation and innate immunity, cholesterol accumulation shows the ability to reinforce macrophages functions through IFN-α regulatory advantages, reprogramming the “set point” between cholesterol synthesis and immune response [127]. Moreover, the M2 isoform of pyruvate kinase (PKM2), a rate-limiting enzyme in the glycolysis pathway, which shows the ability to stimulate HIF-1α transactivation in TAMs and potentially switch the OXPHOS to glycolysis [128], thereby quickly generating sufficient energy in nutritionally competitive and anoxic TME. Meanwhile, HIF-1α-induced miR‐30c/REDD1/mTOR signaling was directly involved in TAMs glycolysis in the hypoxic TME and promoted M1-like polarization [129]. M1-like TAMs produce cytotoxic NO, ROS, and activate Th1 immune responses to carry out tumoricidal activities [130]. Of note, mTOR signaling is an important regulator for glycolysis-cholesterol metabolic axis, which promotes the TAMs repolarization, glycolysis and mevalonate-related cholesterol synthesis [131, 132]. In this aspect, PcrV cultured TAMs into anti-tumor M1 phenotype. The PI3k/Akt/mTOR-glycolysis-NO feedback loop is activated by PcrV, which encourages TAMs repolarization and cytotoxicity against tumor [133].

The upregulated TAMs glycolysis in liver was supposed to promote carbonic anhydrase XII (CA12) in the membrane, thereby inducing a pro-tumoral phenotype in tumor development and metastasis [134]. Glycolysis-induced CA12 can effectively protect macrophages against acidic TME [135]. The increased CA12 mainly depends on the HIF-1α signaling-induced glycolysis activation [135]. Meanwhile, the inhibition of CA12 not only inhibited tumor growth and TAM infiltration, but also decreased TAM-derived CCL8 to improve TME. Meanwhile, a recent study indicated that carbonic anhydrase signaling was involved in cholesterol synthesis, wherein the CA3 in the cytoplasm could interact with the cholesterol rate-limiting enzyme squalene epoxidase (SQLE) and enhance cholesterol synthesis [136]. In some lower cholesterol levels TAMs, cholesterol synthesis and metabolism genes were down-regulated, while cholesterol efflux transporters were up-regulated like ABCA1 and ABCG1 [137]. The efflux transporters induced cholesterol loss supported the M2-like TAMs polarization, and ABCA1 silencing was likely to promote M1 polarizing signaling [109]. Indeed, macrophages have been proved to have intrinsic tumoricidal activity, but they could rapidly adopt an alternative phenotype that leads to tumor immunosuppression [138]. The functional polarization of TAMs and tumor development in vivo are greatly influenced by PI3K/HIF-1α signaling-induced glycolysis-cholesterol metabolic axis, including glycolysis and enhanced cholesterol excretion [109, 112]. Interestingly, in the ID8 mouse model, specific deletion of both ABCA1 and ABCG1 could abolish TAMs cholesterol efflux, and contribute to tumor regression [109]. Moreover, in breast cancer, the cholesterol metabolite 27-hydroxycholesterol (27-HC) can promote the tumor growth and metastasis [139]. In compared with M0 and M1 macrophages, M2-like macrophages produced more 27-HC. The generation of 27-HC in M2-like TAMs is probably caused by the hydroxycholesterol synthesizing enzyme, which can be abrogated by the 27-HC degrading enzyme CYP7B1, subsequently promoting the tumor progress [140]. Previous studies have shown that macrophage lactate dehydrogenase B (LDHB) can convert lactate to pyruvate in malignant cells and TAMs [141]. Frank et al. revealed that macrophage LDHB was down-regulated in both murine and human tumor [18]. The lower LDHB enhanced lactagenesis and glycolysis in TAMs by tumor-derived miR-375, thereby enhancing TAMs cholesterol synthesis [18]. In terms of glycolysis-cholesterol metabolic axis, cholesterol biosynthesis in macrophages is promoted with lactagenesis activated SREBP2. The PI3k/mTOR signaling activated SREBP2 upregulated cholesterol biosynthesis, and was accompanied with increased glucose uptake and lactate secretion [142]. The down-regulation of LDHB skews TAMs to become a source of lactate and sterol/oxysterol for tumor cell proliferation. It is necessary to untangle the cue in regulating glycolysis-cholesterol metabolic axis in TAMs.

### Glycolysis-cholesterol metabolic axis and tumor immunotherapy

Currently, the development of immunotherapy has led to a paradigm shift in tumor treatment. However, inadequate understanding of the immunosuppressive TME metabolic reprogramming prevent majority patients from more effective treatment [27]. Recent studies have shown that immunotherapy can synergize with targeting tumor and/or immune cell metabolism (Table 1). The generally modest response rates to immunotherapies may be improved by comprehending and taking advantage of metabolic interactions in the TME.

**Table 1:** Modulates glycolysis-cholesterol axis-related drugs in combination with immunotherapy

	Glycolysis/cholesterol related drugs	Immune factors	Combined immunological drugs	References
Glycolysis	Non-steroidal anti-inflammatory drug (NSAID)	T cells	Anti-PD1	[64, 143]
	Rapamycin	TAMs	Anti-PD-L1	[131, 133]
	Carbonic anhydrase XII (CA12) inhibitor	Tumor-infiltrating macrophages	Anti-PD-1	[134]
Cholesterol	5-azacytidine	TAMs and T cells	//	[87]
	Simvastatin	PD-L1 expression	Anti-PD-L1	[156]
	LXR agonist	MDSCs	Anti-PD-L1	[94]

### Glycolysis based therapy approach

In adoptive T cells therapy (ACT), glycolysis signaling has the potential to enhance refractory tumor immune resistance, consequently limiting therapeutic effects [31]. The nutritional deprivation, metabolism disorder and complex signaling networks between the tumor and T cells indicated that, in addition to immunosuppression, ACT-refractory tumors and lower T cells-mediated killing might be perturbed by glycolysis-cholesterol metabolic axis-induced cell responses and immune resistance [31, 41]. Ideally, the glycolysis restriction like non-steroidal anti-inflammatory drug (NSAID) administration will disturb glucose metabolism by switching glycolysis into TCA and improving respiration, which will reinforce T-cell effector functions and anti-PD1 treatment [40, 143]. However, the direct link within glycolysis-cholesterol metabolic axis requires further investigation. Through competitive reactivation, the balance between T cells and Tregs in TME implies the anti-PD-1 immunotherapy efficacy [144]. Glycolysis intervention could improve inefficient anti-PD-1 therapy by reversing the disequilibrium between effector T cells and Tregs, thereby achieving safer and more effective immunotherapy [145]. Of note, mTOR signaling acted as a prevailing regulator in glycolysis and underlay the potential candidate for new combination immunotherapies, which may be beneficial for immunotherapy-refractory tumor patients [146]. The anti-PD-L1 and rapamycin co-delivery system efficiently suppressed the tumor glycolysis metabolism, lactate release and M2-like TAMs polarization, as well as improving the immunosuppressive TME and tumor regression [131, 133]. In addition, targeting HIF-1α could potentiate PD-1/PD-L1 immunotherapy by increasing normal tissue immune tolerance and immune-induced tumor regression, as well as reducing immune-related adverse events [147]. This relatively new concept emphasized that HIF-1α signaling is associated with glycolysis-cholesterol metabolic axis intrinsic regulation, thereby fortifying PD-L1 immunotherapy and overcoming immune evasion in the TME [72, 112, 147]. Notably, in macrophages from the hepatocellular carcinoma, HIF-1α-induced CA12 was positively correlated with glycolysis, which showed the ability to promote tumor regression in mice and was sufficient to synergistically enhance the anti-PD-1 therapy effects [134]. The above observation made it possible for glycolysis-cholesterol metabolic axis modulation to be a potential partner for immune checkpoint therapy.

### Cholesterol based therapy approach

The cholesterol regulation has a great potential to ameliorate the TME and guides novel combination immunotherapies against malignant tumor [148]. One hypothesis is that cholesterol is involved in immune cells regulation and renders immune checkpoint therapy more effective [149, 150]. In line with this, high serum cholesterol could enhance NKs anti-tumor effects against hepatocellular carcinoma and alleviate tumor progression by cholesterol-related immunomodulation [151]. Recently, within the TME, dual role of 5-azacytidine in both immune regulation and cholesterol regulation has been concerned. 5-azacytidine could promote TAMs cholesterol accumulation and M1-like polarization, subsequently stimulating effector T cells to attack solid tumors [152]. In addition, 5-azacytidine bind to ATP-binding cassette transporter A9 (ABC-A9) will maintain the intracellular cholesterol homeostasis [152, 153]. The inhibition of TAMs cholesterol efflux and intracellular metabolites contents will rescue the T cells anti-tumor ability [154]. The results of a prospective observational study (UMIN000021694) showed that combination therapy with nivolumab and statins could improve advanced non-small cell lung cancer (NSCLC) outcomes than the nivolumab alone [155]. In colorectal tumor, simvastatin administration not merely perturbs total cholesterol synthesis, but also suppresses PD-L1 expression to enhance anti-tumor immunity [156]. Combining simvastatin with anti-PD-L1 antibodies could potentiate immune checkpoint therapy effects and uncover more immunotherapy strategies [156]. Analogously, the role of LXR agonist-mediated cholesterol metabolism in immunosuppressive MDSCs could reverse un-responsive anti-PD-L1 immunotherapy, and elicit augmented or synergistic anti-tumor immune responses [94]. The cholesterol intervene is an effective strategy to improve TME-induced immune cell exhaustion and enhance immunotherapy like PD-L1-mediated immune evasion [6, 29]. In terms of this, traditional hypercholesterolemia target PCSK9 inhibition also shows the ability to enhance anti-PD-1 effect and CD8^+^ T cells activity in tumor, as well as suppressing the MDSCs infiltration [157, 158]. Although the optimum strategy is likely to differ across different cancer types and patient settings, future study directions could be oriented to assess the effectiveness of "double-whammy" approaches that combine glycolysis-cholesterol metabolic axis and immunosuppressive signatures.

## Conclusion and perspectives

Plasticity and diversity are long-recognized hallmarks of the tumor immune microenvironment. Multiple lines of evidences show that changes in the glycolysis-cholesterol metabolic axis can modulate the tumor immune response via various approaches. Evidently, by responding to intermediate signals, glycolysis-cholesterol metabolic axis in tumor and immune cells has the potential to shape the TME. Although glycolysis and cholesterol metabolism allow the cell to meet their requirements for proliferation and differentiation, these manifestations are likely not the main reason for the harsh tumor milieu. Impairment and suppressive potency of tumor immunity were regulated by a complex milieu of glycolysis-cholesterol metabolic axis mediators. Determination of byproducts and related signaling cascades are important to elucidate the controversial role of the glycolysis-cholesterol metabolic axis in immunosuppressive TME.

Notably, the glycolysis-cholesterol metabolic axis reprograms cells activity and function, consequently leading to the deteriorative TME. The protumor or antitumor roles of immune cells were influenced by conditional glycolysis-cholesterol metabolic axis modulation, cancer immune phenotypes and immunotherapy efficacy. Of note, clinical trials of immunotherapy agents that against a single target did not yield impressive therapeutic efficacy in patients, but metabolic intervention showed the great potential in auxiliary therapy. Although various combinations of metabolic agents and immunotherapies are already applied in clinical trials, efforts to better understand the glycolysis-cholesterol metabolic axis within TME are necessary. The mechanisms of tumor immune modulation are essential to fully exploit the therapeutic potential of combination therapies and improve anti-tumor immunotherapy.

## Data Availability

Not applicable.

## Abbreviations

TME: Tumor microenvironment
KRAS: Kirsten RAt Sarcoma
MYC: Myelocytomatosis
LXR: Liver X receptor
CRAC: Cholesterol-recognition amino acid consensus
Tregs: Regulatory T cells
NKT: Natural killer T-cell
IFN-γ: Interferon-γ
LDLR: Low-density lipoprotein receptor
DCs: Dendritic cells
mcDCs: Merocytic DCs
TLRs: Toll-like receptors
OXPHOS: Oxidative phosphorylation
LXR: Liver X receptor
ApoE: Apolipoprotein E
MDSCs: Myeloid-derived suppressor cells
CEBPB: CCAAT/enhancer-binding protein beta
AMPK: Activated protein kinase
LDHA: Lactic acid dehydrogenase
EBV: Epstein-Barr virus
LMP1: Latent membrane protein 1
β2-AR: β2-Adrenergic receptor
FAO: Fatty acid oxidation
TAMs: Tumor-associated macrophages
PKM2: Pyruvate kinase
CA12: Carbonic anhydrase XII
SQLE: Squalene epoxidase
ACT: Adoptive T-cell therapy
NSAID: Non-steroidal anti-inflammatory drug
NSCLC: Non-small cell lung cancer

## References

[1] Ghesquière B, Wong BW, Kuchnio A, Carmeliet P (2014). Metabolism of stromal and immune cells in health and disease. Nature.

[2] Bull CJ, Bell JA, Murphy N, Sanderson E, Davey Smith G, Timpson NJ (2020). Adiposity, metabolites, and colorectal cancer risk: Mendelian randomization study. BMC Med.

[3] Koop AC, Bossers GPL, Ploegstra MJ, Hagdorn QAJ, Berger RMF, Silljé HHW (2019). Metabolic remodeling in the pressure-loaded right ventricle: shifts in glucose and fatty acid metabolism-a systematic review and meta-analysis. J Am Heart Assoc.

[4] Zhang J, Xiao X, Guo Q, Wei Z, Hua W (2022). Identification of four metabolic subtypes of glioma based on glycolysis-cholesterol synthesis genes. Comput Math Methods Med.

[5] Xia L, Oyang L, Lin J, Tan S, Han Y, Wu N (2021). The cancer metabolic reprogramming and immune response. Mol Cancer.

[6] Ma X, Bi E, Lu Y, Su P, Huang C, Liu L (2019). Cholesterol induces CD8 (+) T cell exhaustion in the tumor microenvironment. Cell Metab.

[7] Ceroi A, Masson D, Roggy A, Roumier C, Chagué C, Gauthier T (2016). LXR agonist treatment of blastic plasmacytoid dendritic cell neoplasm restores cholesterol efflux and triggers apoptosis. Blood.

[8] Li X, Wenes M, Romero P, Huang SC, Fendt SM, Ho PC (2019). Navigating metabolic pathways to enhance antitumour immunity and immunotherapy. Nat Rev Clin Oncol.

[9] DeBerardinis RJ, Chandel NS (2016). Fundamentals of cancer metabolism. Sci Adv.

[10] Huang B, Song BL, Xu C (2020). Cholesterol metabolism in cancer: mechanisms and therapeutic opportunities. Nat Metab.

[11] Liu Z, Luo Y, Ren J, Yang L, Li J, Wei Z (2022). Association between fish oil supplementation and cancer risk according to fatty fish consumption: A large prospective population-based cohort study using UK Biobank. Int J Cancer.

[12] Mitroulis I, Ruppova K, Wang B, Chen LS, Grzybek M, Grinenko T (2018). Modulation of myelopoiesis progenitors is an integral component of trained immunity. Cell.

[13] Karasinska JM, Topham JT, Kalloger SE, Jang GH, Denroche RE, Culibrk L (2020). Altered gene expression along the glycolysis-cholesterol synthesis axis is associated with outcome in pancreatic cancer. Clinical Cancer Res.

[14] Zhong PC, Shu R, Wu HW, Liu ZW, Shen XL, Hu YJ (2021). Altered gene expression in glycolysis-cholesterol synthesis axis correlates with outcome of triple-negative breast cancer. Exp Biol Med.

[15] Icard P, Wu Z, Fournel L, Coquerel A, Lincet H, Alifano M (2020). ATP citrate lyase: a central metabolic enzyme in cancer. Cancer Lett.

[16] Hunt BG, Davis JC, Fox LH, Vicente-Muñoz S, Lester C, Wells SI (2023). RON-augmented cholesterol biosynthesis in breast cancer metastatic progression and recurrence. Oncogene.

[17] Wei X, Michelakos T, He Q, Wang X, Chen Y, Kontos F (2023). Association of tumor cell metabolic subtype and immune response with the clinical course of hepatocellular carcinoma. Oncologist.

[18] Frank AC, Raue R, Fuhrmann DC, Sirait-Fischer E, Reuse C, Weigert A (2021). Lactate dehydrogenase B regulates macrophage metabolism in the tumor microenvironment. Theranostics.

[19] Wculek SK, Heras-Murillo I, Mastrangelo A, Mañanes D, Galán M, Miguel V (2023). Oxidative phosphorylation selectively orchestrates tissue macrophage homeostasis. Immunity.

[20] Huang J, Zhao X, Li X, Peng J, Yang W, Mi S (2021). HMGCR inhibition stabilizes the glycolytic enzyme PKM2 to support the growth of renal cell carcinoma. PLoS Biol.

[21] Gautier EL, Westerterp M, Bhagwat N, Cremers S, Shih A, Abdel-Wahab O (2013). HDL and Glut1 inhibition reverse a hypermetabolic state in mouse models of myeloproliferative disorders. J Exp Med.

[22] Takahashi H, Kawabata-Iwakawa R, Ida S, Mito I, Tada H, Chikamatsu K (2021). Upregulated glycolysis correlates with tumor progression and immune evasion in head and neck squamous cell carcinoma. Sci Rep.

[23] Jiang J, Zheng Q, Zhu W, Chen X, Lu H, Chen D (2020). Alterations in glycolytic/cholesterogenic gene expression in hepatocellular carcinoma. Aging.

[24] Liu PS, Chen YT, Li X, Hsueh PC, Tzeng SF, Chen H (2023). CD40 signal rewires fatty acid and glutamine metabolism for stimulating macrophage anti-tumorigenic functions. Nat Immunol.

[25] Lunt SY, Vander Heiden MG (2011). Aerobic glycolysis: meeting the metabolic requirements of cell proliferation. Annu Rev Cell Dev Biol.

[26] Bonacina F, Coe D, Wang G, Longhi MP, Baragetti A, Moregola A (2018). Myeloid apolipoprotein E controls dendritic cell antigen presentation and T cell activation. Nat Commun.

[27] Ping Y, Shen C, Huang B, Zhang Y (2022). Reprogramming T-Cell metabolism for better anti-tumor immunity. Cells.

[28] Chang CH, Qiu J, O'Sullivan D, Buck MD, Noguchi T, Curtis JD (2015). Metabolic competition in the tumor microenvironment is a driver of cancer progression. Cell.

[29] Wang Q, Cao Y, Shen L, Xiao T, Cao R, Wei S (2022). Regulation of PD-L1 through direct binding of cholesterol to CRAC motifs. Sci Adv.

[30] Coleman PS, Parlo RA (2021). Warburg's ghost-cancer's self-sustaining phenotype: the aberrant carbon flux in cholesterol-enriched tumor mitochondria via deregulated cholesterogenesis. Front Cell Develop Biol.

[31] Cascone T, McKenzie JA, Mbofung RM, Punt S, Wang Z, Xu C (2018). Increased tumor glycolysis characterizes immune resistance to adoptive T cell therapy. Cell Metab.

[32] Wang G, Wang JJ, Guan R, Sun Y, Shi F, Gao J (2019). Targeting strategies for glucose metabolic pathways and T cells in colorectal cancer. Curr Cancer Drug Targets.

[33] Gumus R, Capik O, Gundogdu B, Tatar A, Altinkaynak K, Ozdemir Tozlu O (2021). Low vitamin D and high cholesterol facilitate oral carcinogenesis in 4NQO-induced rat models via regulating glycolysis. Oral Dis.

[34] Niu D, Luo T, Wang H, Xia Y, Xie Z (2021). Lactic acid in tumor invasion. Clinica Chimica Acta Int J Clin Chem..

[35] Rostamian H, Khakpoor-Koosheh M, Jafarzadeh L, Masoumi E, Fallah-Mehrjardi K, Tavassolifar MJ (2022). Restricting tumor lactic acid metabolism using dichloroacetate improves T cell functions. BMC Cancer.

[36] Tu VY, Ayari A, O'Connor RS (2021). Beyond the lactate paradox: how lactate and acidity impact T cell therapies against cancer. Antibodies.

[37] Deng H, Kan A, Lyu N, He M, Huang X, Qiao S (2021). Tumor-derived lactate inhibit the efficacy of lenvatinib through regulating PD-L1 expression on neutrophil in hepatocellular carcinoma. J Immunother Cancer.

[38] Kumagai S, Koyama S, Itahashi K, Tanegashima T, Lin YT, Togashi Y (2022). Lactic acid promotes PD-1 expression in regulatory T cells in highly glycolytic tumor microenvironments. Cancer Cell.

[39] Li W, Xu M, Li Y, Huang Z, Zhou J, Zhao Q (2020). Comprehensive analysis of the association between tumor glycolysis and immune/inflammation function in breast cancer. J Transl Med.

[40] Renner K, Bruss C, Schnell A, Koehl G, Becker HM, Fante M (2019). Restricting glycolysis preserves T cell effector functions and augments checkpoint therapy. Cell Rep.

[41] Fu S, He K, Tian C, Sun H, Zhu C, Bai S (2020). Impaired lipid biosynthesis hinders anti-tumor efficacy of intratumoral iNKT cells. Nat Commun.

[42] Oh JH, Hur W, Li N, Jo SJ (2022). Effects of the epidermal growth factor receptor inhibitor, gefitinib, on lipid and hyaluronic acid synthesis in cultured HaCaT keratinocytes. Exp Dermatol.

[43] Morey P, Pfannkuch L, Pang E, Boccellato F, Sigal M, Imai-Matsushima A (2018). Helicobacter pylori depletes cholesterol in gastric glands to prevent interferon gamma signaling and escape the inflammatory response. Gastroenterology.

[44] Cheng Y, Wang D, Jiang J, Huang W, Li D, Luo J (2020). Integrative analysis of AR-mediated transcriptional regulatory network reveals IRF1 as an inhibitor of prostate cancer progression. Prostate.

[45] Dong L, Yang X, Wang Y, Jin Y, Zhou Q, Chen G (2021). Key markers involved in the anticolon cancer response of CD8+ T cells through the regulation of cholesterol metabolism. J Oncol.

[46] Yang W, Bai Y, Xiong Y, Zhang J, Chen S, Zheng X (2016). Potentiating the antitumour response of CD8 (+) T cells by modulating cholesterol metabolism. Nature.

[47] Menk AV, Scharping NE, Moreci RS, Zeng X, Guy C, Salvatore S (2018). Early TCR signaling induces rapid aerobic glycolysis enabling distinct acute T cell effector functions. Cell Rep.

[48] Zhu M, Zhao X, Chen J, Xu J, Hu G, Guo D (2015). ACAT1 regulates the dynamics of free cholesterols in plasma membrane which leads to the APP-α-processing alteration. Acta Biochim Biophys Sin.

[49] Yuan J, Cai T, Zheng X, Ren Y, Qi J, Lu X (2021). Potentiating CD8 (+) T cell antitumor activity by inhibiting PCSK9 to promote LDLR-mediated TCR recycling and signaling. Protein Cell.

[50] McKinney EF, Smith KGC (2018). Metabolic exhaustion in infection, cancer and autoimmunity. Nat Immunol.

[51] Siska PJ, van der Windt GJ, Kishton RJ, Cohen S, Eisner W, MacIver NJ (2016). Suppression of Glut1 and glucose metabolism by decreased Akt/mTORC1 signaling drives T cell impairment in B cell leukemia. J immunol.

[52] Xu J, Dang Y, Ren YR, Liu JO (2010). Cholesterol trafficking is required for mTOR activation in endothelial cells. Proc Natl Acad Sci USA.

[53] Greco B, Malacarne V, De Girardi F, Scotti GM, Manfredi F, Angelino E (2022). Disrupting N-glycan expression on tumor cells boosts chimeric antigen receptor T cell efficacy against solid malignancies. Sci Transl Med..

[54] Lei K, Kurum A, Kaynak M, Bonati L, Han Y, Cencen V (2021). Cancer-cell stiffening via cholesterol depletion enhances adoptive T-cell immunotherapy. Nat Biomed Eng.

[55] Giovanelli P, Sandoval TA, Cubillos-Ruiz JR (2019). Dendritic cell metabolism and function in tumors. Trends Immunol.

[56] Krawczyk CM, Holowka T, Sun J, Blagih J, Amiel E, DeBerardinis RJ (2010). Toll-like receptor-induced changes in glycolytic metabolism regulate dendritic cell activation. Blood.

[57] Audiger C, Fois A, Thomas AL, Janssen E, Pelletier M, Lesage S (2020). Merocytic dendritic cells compose a conventional dendritic cell subset with low metabolic activity. J Immunol.

[58] Barton GM, Medzhitov R (2002). Control of adaptive immune responses by Toll-like receptors. Curr Opin Immunol.

[59] Brück J, Pascolo S, Fuchs K, Kellerer C, Glocova I, Geisel J (2015). Cholesterol modification of p40-specific small interfering RNA enables therapeutic targeting of dendritic cells. J Immunol.

[60] Everts B, Amiel E, van der Windt GJ, Freitas TC, Chott R, Yarasheski KE (2012). Commitment to glycolysis sustains survival of NO-producing inflammatory dendritic cells. Blood.

[61] de Winde CM, Munday C, Acton SE (2020). Molecular mechanisms of dendritic cell migration in immunity and cancer. Med Microbiol Immunol.

[62] Guak H, Al Habyan S, Ma EH, Aldossary H, Al-Masri M, Won SY (2018). Glycolytic metabolism is essential for CCR7 oligomerization and dendritic cell migration. Nat Commun.

[63] Fontana R, Paniccia A, Russo V (2016). Detection and functional analysis of tumor-derived LXR ligands. Methods Mol Biol.

[64] Liu J, Zhang X, Chen K, Cheng Y, Liu S, Xia M (2019). CCR7 chemokine receptor-inducible lnc-dpf3 restrains dendritic cell migration by inhibiting HIF-1α-mediated glycolysis. Immunity.

[65] Nazih H, Bard JM (2020). Cholesterol, oxysterols and LXRs in breast cancer pathophysiology. Int J Mol Sci.

[66] Jakobsson T, Treuter E, Gustafsson J, Steffensen KR (2012). Liver X receptor biology and pharmacology: new pathways, challenges and opportunities. Trends Pharmacol Sci.

[67] Hanley TM, Blay Puryear W, Gummuluru S, Viglianti GA (2010). PPARgamma and LXR signaling inhibit dendritic cell-mediated HIV-1 capture and trans-infection. PLoS Pathog.

[68] Strauss L, Mahmoud MAA, Weaver JD, Tijaro-Ovalle NM, Christofides A, Wang Q (2020). Targeted deletion of PD-1 in myeloid cells induces antitumor immunity. Science immunology..

[69] Zevini A, Palermo E, Di Carlo D, Alexandridi M, Rinaldo S, Paone A (2022). Inhibition of glycolysis impairs retinoic acid-inducible gene I-mediated antiviral responses in primary human dendritic cells. Front Cell Infect Microbiol.

[70] Sun Y, Zhou L, Chen W, Zhang L, Zeng H, Sun Y (2022). Immune metabolism: a bridge of dendritic cells function. Int Rev Immunol.

[71] Du X, Chapman NM, Chi H (2018). Emerging roles of cellular metabolism in regulating dendritic cell subsets and function. Front Cell Develop Biol.

[72] Thomas C, Leleu D, Masson D (2022). Cholesterol and HIF-1α: dangerous liaisons in atherosclerosis. Front Immunol.

[73] O'Rourke SA, Neto NGB, Devilly E, Shanley LC, Fitzgerald HK, Monaghan MG (2022). Cholesterol crystals drive metabolic reprogramming and M1 macrophage polarisation in primary human macrophages. Atherosclerosis.

[74] Jin X, Zhang W, Wang Y, Liu J, Hao F, Li Y (2020). Pyruvate Kinase M2 promotes the activation of dendritic cells by enhancing IL-12p35 Expression. Cell Rep.

[75] Geng G, Xu C, Peng N, Li Y, Liu J, Wu J (2021). PTBP1 is necessary for dendritic cells to regulate T-cell homeostasis and antitumour immunity. Immunology.

[76] Zhu Y, Liu Z, Wan Y, Zou L, Liu L, Ding S (2022). PARP14 promotes the growth and glycolysis of acute myeloid leukemia cells by regulating HIF-1α expression. Clinical Immunol.

[77] Sarrazy V, Viaud M, Westerterp M, Ivanov S, Giorgetti-Peraldi S, Guinamard R (2016). Disruption of Glut1 in hematopoietic stem cells prevents myelopoiesis and enhanced glucose flux in atheromatous plaques of ApoE (-/-) Mice. Circ Res.

[78] Leeman H, Kaminska E, Green D, Bodman-Smith M, Gravett A, Bodman-Smith K (2019). Serum apolipoprotein E and other inflammatory markers can identify non-responding patients to a dendritic cell vaccine. Translat Oncol.

[79] Hu C, Pang B, Lin G, Zhen Y, Yi H (2020). Energy metabolism manipulates the fate and function of tumour myeloid-derived suppressor cells. Br J Cancer.

[80] Cai TT, Ye SB, Liu YN, He J, Chen QY, Mai HQ (2017). LMP1-mediated glycolysis induces myeloid-derived suppressor cell expansion in nasopharyngeal carcinoma. PLoS Pathog.

[81] Tavazoie MF, Pollack I, Tanqueco R, Ostendorf BN, Reis BS, Gonsalves FC (2018). LXR agonism depletes MDSCs to promote antitumor immunity. Cancer Discov.

[82] Parker KH, Beury DW, Ostrand-Rosenberg S (2015). Myeloid-derived suppressor cells: critical cells driving immune suppression in the tumor microenvironment. Adv Cancer Res.

[83] Sun HW, Wu WC, Chen HT, Xu YT, Yang YY, Chen J (2020). Glutamine deprivation promotes the generation and mobilization of MDSCs by enhancing expression of G-CSF and GM-CSF. Front Immunol.

[84] Li W, Tanikawa T, Kryczek I, Xia H, Li G, Wu K (2018). Aerobic glycolysis controls myeloid-derived suppressor cells and tumor immunity via a specific CEBPB isoform in triple-negative breast cancer. Cell Metab.

[85] Krishan S, Sahni S, Leck LYW, Jansson PJ, Richardson DR (2020). Regulation of autophagy and apoptosis by Dp44mT-mediated activation of AMPK in pancreatic cancer cells. Biochim Biophys Acta.

[86] Kim J, Kundu M, Viollet B, Guan KL (2011). AMPK and mTOR regulate autophagy through direct phosphorylation of Ulk1. Nat Cell Biol.

[87] Shi S, Ji X, Shi J, Shi S, She F, Zhang Q (2022). Andrographolide in atherosclerosis: integrating network pharmacology and in vitro pharmacological evaluation. Biosci Rep.

[88] Jian SL, Chen WW, Su YC, Su YW, Chuang TH, Hsu SC (2017). Glycolysis regulates the expansion of myeloid-derived suppressor cells in tumor-bearing hosts through prevention of ROS-mediated apoptosis. Cell Death Dis.

[89] Fu C, Fu Z, Jiang C, Xia C, Zhang Y, Gu X (2021). CD205 (+) polymorphonuclear myeloid-derived suppressor cells suppress antitumor immunity by overexpressing GLUT3. Cancer Sci.

[90] Fonseca P, Vardaki I, Occhionero A, Panaretakis T (2016). Metabolic and signaling functions of cancer cell-derived extracellular vesicles. Int Rev Cell Mol Biol.

[91] Rincón-Riveros A, Lopez L, Villegas EV, Antonia RJ (2021). Regulation of antitumor immune responses by exosomes derived from tumor and immune cells. Cancers.

[92] Meckes DG, Menaker NF, Raab-Traub N (2013). Epstein-Barr virus LMP1 modulates lipid raft microdomains and the vimentin cytoskeleton for signal transduction and transformation. J Virol.

[93] Maenhout SK, Van Lint S, Emeagi PU, Thielemans K, Aerts JL (2014). Enhanced suppressive capacity of tumor-infiltrating myeloid-derived suppressor cells compared with their peripheral counterparts. Int J Cancer.

[94] Tavazoie MF, Pollack I, Tanqueco R, Ostendorf BN, Reis BS, Gonsalves FC (2018). LXR/ApoE activation restricts innate immune suppression in cancer. Cell.

[95] Liang H, Shen X (2020). LXR activation radiosensitizes non-small cell lung cancer by restricting myeloid-derived suppressor cells. Biochem Biophys Res Commun.

[96] Ito J, Nagayasu Y, Miura Y, Yokoyama S, Michikawa M (2014). Astrocyte׳s endogenous apoE generates HDL-like lipoproteins using previously synthesized cholesterol through interaction with ABCA1. Brain Res.

[97] Kemp SB, Carpenter ES, Steele NG, Donahue KL, Nwosu ZC, Pacheco A (2021). Apolipoprotein E promotes immune suppression in pancreatic cancer through NF-κB-Mediated production of CXCL1. Can Res.

[98] Li B, Lian M, Li Y, Qian Q, Zhang J, Liu Q (2021). Myeloid-derived suppressive cells deficient in liver X receptor α protected from autoimmune hepatitis. Front Immunol.

[99] Waight JD, Netherby C, Hensen ML, Miller A, Hu Q, Liu S (2013). Myeloid-derived suppressor cell development is regulated by a STAT/IRF-8 axis. J Clin Investig.

[100] Chauhan KS, Das A, Jaiswal H, Saha I, Kaushik M, Patel VK (2022). IRF8 and BATF3 interaction enhances the cDC1 specific Pfkfb3 gene expression. Cell Immunol.

[101] Adorni MP, Cipollari E, Favari E, Zanotti I, Zimetti F, Corsini A (2017). Inhibitory effect of PCSK9 on Abca1 protein expression and cholesterol efflux in macrophages. Atherosclerosis.

[102] Kumar A, Gupta P, Rana M, Chandra T, Dikshit M, Barthwal MK (2020). Role of pyruvate kinase M2 in oxidized LDL-induced macrophage foam cell formation and inflammation. J Lipid Res.

[103] Li C, You X, Xu X, Wu B, Liu Y, Tong T (2022). A metabolic reprogramming amino acid polymer as an immunosurveillance activator and leukemia targeting drug carrier for T-cell acute lymphoblastic leukemia. Adv Sci.

[104] Mohammadpour H, MacDonald CR, McCarthy PL, Abrams SI, Repasky EA (2021). β2-adrenergic receptor signaling regulates metabolic pathways critical to myeloid-derived suppressor cell function within the TME. Cell Rep.

[105] Ursan R, Odnoshivkina UG, Petrov AM (2020). Membrane cholesterol oxidation downregulates atrial β-adrenergic responses in ROS-dependent manner. Cell Signal.

[106] Cang X, Yang L, Yang J, Luo C, Zheng M, Yu K (2014). Cholesterol-β1 AR interaction versus cholesterol-β2 AR interaction. Proteins.

[107] Netea-Maier RT, Smit JWA, Netea MG (2018). Metabolic changes in tumor cells and tumor-associated macrophages: a mutual relationship. Cancer Lett.

[108] Zheng X, Mansouri S, Krager A, Grimminger F, Seeger W, Pullamsetti SS (2020). Metabolism in tumour-associated macrophages: a quid pro quo with the tumour microenvironment. Euro Respirat Rev..

[109] Goossens P, Rodriguez-Vita J, Etzerodt A, Masse M, Rastoin O, Gouirand V (2019). Membrane cholesterol efflux drives tumor-associated macrophage reprogramming and tumor progression. Cell Metab.

[110] Na KJ, Choi H, Oh HR, Kim YH, Lee SB, Jung YJ (2020). Reciprocal change in glucose metabolism of cancer and immune cells mediated by different glucose transporters predicts immunotherapy response. Theranostics.

[111] Paolini L, Adam C, Beauvillain C, Preisser L, Blanchard S, Pignon P (2020). Lactic acidosis together with GM-CSF and M-CSF induces human macrophages toward an inflammatory protumor phenotype. Cancer Immunol Res.

[112] Jiang H, Wei H, Wang H, Wang Z, Li J, Ou Y (2022). Zeb1-induced metabolic reprogramming of glycolysis is essential for macrophage polarization in breast cancer. Cell Death Dis.

[113] Hu X, Chao M, Wu H (2017). Central role of lactate and proton in cancer cell resistance to glucose deprivation and its clinical translation. Signal Transduct Target Ther.

[114] Geeraerts X, Fernández-Garcia J, Hartmann FJ, de Goede KE, Martens L, Elkrim Y (2021). Macrophages are metabolically heterogeneous within the tumor microenvironment. Cell Rep.

[115] Chen F, Chen J, Yang L, Liu J, Zhang X, Zhang Y (2019). Extracellular vesicle-packaged HIF-1α-stabilizing lncRNA from tumour-associated macrophages regulates aerobic glycolysis of breast cancer cells. Nat Cell Biol.

[116] He C, Hu X, Weston TA, Jung RS, Sandhu J, Huang S (2018). Macrophages release plasma membrane-derived particles rich in accessible cholesterol. Proc Natl Acad Sci USA.

[117] Xiang Y, Miao H (2021). Lipid metabolism in tumor-associated macrophages. Adv Exp Med Biol.

[118] de Brito NM, Duncan Moretti J, da Costa HC, Saldanha Gama R, Paula Neto HA, Dorighello GG (2020). Aerobic glycolysis is a metabolic requirement to maintain the M2-like polarization of tumor-associated macrophages. Biochimica et biophysica acta Mol Cell Res..

[119] Sousa S, Määttä J (2016). The role of tumour-associated macrophages in bone metastasis. J Bone Oncol.

[120] De Palma M, Biziato D, Petrova TV (2017). Microenvironmental regulation of tumour angiogenesis. Nat Rev Cancer.

[121] He Z, Chen D, Wu J, Sui C, Deng X, Zhang P (2021). Yes associated protein 1 promotes resistance to 5-fluorouracil in gastric cancer by regulating GLUT3-dependent glycometabolism reprogramming of tumor-associated macrophages. Arch Biochem Biophys.

[122] Li L, Liu B, Håversen L, Lu E, Magnusson LU, Ståhlman M (2012). The importance of GLUT3 for de novo lipogenesis in hypoxia-induced lipid loading of human macrophages. PLoS ONE.

[123] Freemerman AJ, Johnson AR, Sacks GN, Milner JJ, Kirk EL, Troester MA (2014). Metabolic reprogramming of macrophages: glucose transporter 1 (GLUT1)-mediated glucose metabolism drives a proinflammatory phenotype. J Biol Chem.

[124] Penny HL, Sieow JL, Gun SY, Lau MC, Lee B, Tan J (2021). Targeting glycolysis in macrophages confers protection against pancreatic ductal adenocarcinoma. Int J Mol Sci.

[125] Rezende L, Couto NFD, Fernandes-Braga W, Epshtein Y, Alvarez-Leite JI, Levitan I (2022). OxLDL induces membrane structure rearrangement leading to biomechanics alteration and migration deficiency in macrophage. Biochim Biophys Acta.

[126] Lee SJ, Thien Quach CH, Jung KH, Paik JY, Lee JH, Park JW (2014). Oxidized low-density lipoprotein stimulates macrophage 18F-FDG uptake via hypoxia-inducible factor-1α activation through Nox2-dependent reactive oxygen species generation. J Nucl Med.

[127] York AG, Williams KJ, Argus JP, Zhou QD, Brar G, Vergnes L (2015). Limiting cholesterol biosynthetic flux spontaneously engages type I IFN signaling. Cell.

[128] Palsson-McDermott EM, Dyck L, Zasłona Z, Menon D, McGettrick AF, Mills KHG (2017). Pyruvate kinase M2 Is required for the expression of the immune checkpoint PD-L1 in immune cells and tumors. Front Immunol.

[129] Zhihua Y, Yulin T, Yibo W, Wei D, Yin C, Jiahao X (2019). Hypoxia decreases macrophage glycolysis and M1 percentage by targeting microRNA-30c and mTOR in human gastric cancer. Cancer Sci.

[130] Pathria P, Louis TL, Varner JA (2019). Targeting tumor-associated macrophages in cancer. Trends Immunol.

[131] Chen B, Gao A, Tu B, Wang Y, Yu X, Wang Y (2020). Metabolic modulation via mTOR pathway and anti-angiogenesis remodels tumor microenvironment using PD-L1-targeting codelivery. Biomaterials.

[132] Kang J, Lee D, Lee KJ, Yoon JE, Kwon JH, Seo Y (2022). Tumor-suppressive effect of metformin via the regulation of m2 macrophages and myeloid-derived suppressor cells in the tumor microenvironment of colorectal cancer. Cancers.

[133] Yu H, Bai Y, Qiu J, He X, Xiong J, Dai Q (2021). Pseudomonas aeruginosa PcrV Enhances the Nitric Oxide-Mediated Tumoricidal Activity of Tumor-Associated Macrophages via a TLR4/PI3K/AKT/mTOR-Glycolysis-Nitric Oxide Circuit. Front Oncol.

[134] Ning WR, Jiang D, Liu XC, Huang YF, Peng ZP, Jiang ZZ (2022). Carbonic anhydrase XII mediates the survival and prometastatic functions of macrophages in human hepatocellular carcinoma. J Clin Invest.

[135] Graham N, Pollard JW (2022). An acid trip activates protumoral macrophages to promote hepatocellular carcinoma malignancy. J Clin Invest.

[136] Liu D, Wong CC, Zhou Y, Li C, Chen H, Ji F (2021). Squalene epoxidase induces nonalcoholic steatohepatitis via binding to carbonic anhydrase III and is a therapeutic target. Gastroenterology.

[137] Hoppstädter J, Dembek A, Höring M, Schymik HS, Dahlem C, Sultan A (2021). Dysregulation of cholesterol homeostasis in human lung cancer tissue and tumour-associated macrophages. EBioMedicine.

[138] Hagemann T, Lawrence T, McNeish I, Charles KA, Kulbe H, Thompson RG (2008). "Re-educating" tumor-associated macrophages by targeting NF-kappaB. J Exp Med.

[139] Shen Z, Zhu D, Liu J, Chen J, Liu Y, Hu C (2017). 27-Hydroxycholesterol induces invasion and migration of breast cancer cells by increasing MMP9 and generating EMT through activation of STAT-3. Environ Toxicol Pharmacol.

[140] Shi SZ, Lee EJ, Lin YJ, Chen L, Zheng HY, He XQ (2019). Recruitment of monocytes and epigenetic silencing of intratumoral CYP7B1 primarily contribute to the accumulation of 27-hydroxycholesterol in breast cancer. Am J Cancer Res.

[141] McCleland ML, Adler AS, Shang Y, Hunsaker T, Truong T, Peterson D (2012). An integrated genomic screen identifies LDHB as an essential gene for triple-negative breast cancer. Can Res.

[142] Misra UK, Pizzo SV (2015). Activated α2-macroglobulin binding to human prostate cancer cells triggers insulin-like responses. J Biol Chem.

[143] Singer K, Dettmer K, Unger P, Schönhammer G, Renner K, Peter K (2019). Topical diclofenac reprograms metabolism and immune cell infiltration in actinic keratosis. Front Oncol.

[144] Yoshida K, Okamoto M, Sasaki J, Kuroda C, Ishida H, Ueda K (2020). Anti-PD-1 antibody decreases tumour-infiltrating regulatory T cells. BMC Cancer.

[145] Geng Z, Dong B, Lv W, Wang Z, Wang X, Huang Y (2022). LncRNA ZFAS1 regulates the proliferation, oxidative stress, fibrosis, and inflammation of high glucose-induced human mesangial cells via the miR-588/ROCK1 axis. Diabetol Metab Syndr.

[146] Conciatori F, Bazzichetto C, Falcone I, Pilotto S, Bria E, Cognetti F (2018). Role of mTOR signaling in tumor microenvironment: an overview. Int J Mol Sci.

[147] Bailey CM, Liu Y, Liu M, Du X, Devenport M, Zheng P (2022). Targeting HIF-1α abrogates PD-L1-mediated immune evasion in tumor microenvironment but promotes tolerance in normal tissues. J Clin Invest.

[148] King RJ, Singh PK, Mehla K (2022). The cholesterol pathway: impact on immunity and cancer. Trends Immunol.

[149] Perrone F, Minari R, Bersanelli M, Bordi P, Tiseo M, Favari E (2020). The prognostic role of high blood cholesterol in advanced cancer patients treated with immune checkpoint inhibitors. J Immunother.

[150] McDermott DF, Huseni MA, Atkins MB, Motzer RJ, Rini BI, Escudier B (2018). Clinical activity and molecular correlates of response to atezolizumab alone or in combination with bevacizumab versus sunitinib in renal cell carcinoma. Nat Med.

[151] Qin WH, Yang ZS, Li M, Chen Y, Zhao XF, Qin YY (2020). High serum levels of cholesterol increase antitumor functions of nature killer cells and reduce growth of liver tumors in mice. Gastroenterology.

[152] Shi R, Zhao K, Wang T, Yuan J, Zhang D, Xiang W (2022). 5-aza-2'-deoxycytidine potentiates anti-tumor immunity in colorectal peritoneal metastasis by modulating ABC A9-mediated cholesterol accumulation in macrophages. Theranostics.

[153] Plummer AM, Culbertson AT, Liao M (2021). The ABCs of Sterol Transport. Annu Rev Physiol.

[154] Wang S, Yan W, Kong L, Zuo S, Wu J, Zhu C (2023). Oncolytic viruses engineered to enforce cholesterol efflux restore tumor-associated macrophage phagocytosis and anti-tumor immunity in glioblastoma. Nat Commun.

[155] Omori M, Okuma Y, Hakozaki T, Hosomi Y (2019). Statins improve survival in patients previously treated with nivolumab for advanced non-small cell lung cancer: an observational study. Mol Clin Oncol.

[156] Ni W, Mo H, Liu Y, Xu Y, Qin C, Zhou Y (2021). Targeting cholesterol biosynthesis promotes anti-tumor immunity by inhibiting long noncoding RNA SNHG29-mediated YAP activation. Mol Therapy J Am Soc Gene Therapy.

[157] Yang QC, Wang S, Liu YT, Song A, Wu ZZ, Wan SC (2023). Targeting PCSK9 reduces cancer cell stemness and enhances antitumor immunity in head and neck cancer. iScience..

[158] Masuda Y, Yamaguchi S, Suzuki C, Aburatani T, Nagano Y, Miyauchi R (2018). Generation and characterization of a novel small biologic alternative to proprotein convertase subtilisin/kexin type 9 (PCSK9) antibodies, DS-9001a, albumin binding domain-fused anticalin protein. J Pharmacol Exp Ther.

